# Large-Scale Analyses of GWAS Identify Five Key Pleiotropic Genes Involved in Complex Diseases

**DOI:** 10.3390/genes17070766

**Published:** 2026-06-30

**Authors:** Imene Mahdi, Ghazi Chabchoub, Najla Kharrat, Ahmed Rebai

**Affiliations:** 1 Laboratory of Molecular and Cellular Screening Processes, Centre of Biotechnology of Sfax, University of Sfax, P.O. Box 1177, Sfax 3018, Tunisia; imene.mahdi@yahoo.fr (I.M.);; 2 Laboratory of Human Molecular Genetics, Faculty of Medicine of Sfax, University of Sfax, Sfax 3018, Tunisia

**Keywords:** pleiotropy, GWAS, complex diseases, ABO, GCKR, TERT

## Abstract

**Background/Objectives**: Genome-Wide Association Studies (GWASs) have revealed numerous Single Nucleotide Polymorphisms (SNPs) linked to diverse diseases and traits, highlighting extensive pleiotropy. This study aimed to identify functionally relevant pleiotropic genes through large-scale analysis of the GWAS catalog and variant annotation. **Methods**: An initial set of 494 putative pleiotropic genes across 223 phenotypes was refined using stringent criteria, yielding 343 SNP–trait associations corresponding to 53 SNPs mapped to 16 genes. **Results**: Five top-ranked genes were prioritized using a composite score, and their variants were fully annotated for genetic and clinical features. Approximately 70% of the SNPs were intronic, with five located in UTRs, and 11 in coding regions. ABO emerged as the highest-scoring pleiotropic gene, with six SNPs in strong linkage disequilibrium, implicating rs8176719 (c.261delG in exon 6) as the likely causal variant. ALDH2 showed 20 strong associations across two SNPs, with rs671 (E504K) identified as a key missense variant linked to multiple diseases. GCKR exhibited 29 associations across three SNPs, with rs1260326 (P446L) reducing glucokinase inhibition and enhancing hepatic glycolysis and triglyceride production. In HLA-DQA1, three of four SNPs located in UTRs suggested regulatory roles in gene expression. TERT displayed 59 GWAS signals, primarily cancer-related across seven organs, involving eight SNPs. Among these, rs10069690 in intron 4 has been associated with altered gene expression, while the functional impact of other variants remains to be clarified. **Conclusions**: This work highlights key pleiotropic genes and variants, offering insights into the genetic architecture and mechanisms underlying complex human diseases.

## 1. Introduction

Genome-wide association studies (GWASs) have significantly advanced our understanding of the genetic basis of human diseases and complex traits, through the identification of thousands of genetic variants associated with a wide array of phenotypes, ranging from common diseases to behavioral traits [1,2].

One of the most important discoveries that emerged from these studies is the unexpected extent of pleiotropy, where genetic variants in a single gene (and sometimes the same variant) are associated to multiple traits or diseases [3,4,5]. This pleiotropic nature complicates the interpretation of GWAS results, but it also provides valuable insights into the genetic architecture of human biology and diseases [3,6,7].

In recent years, with the increasing availability of GWAS summary statistics, genetic pleiotropy appears to be common, if not ubiquitous, in the human genome; nearly half of the genes reported in the GWAS catalog appear to be pleiotropic.

Early studies suggested that pleiotropy might be universal, with genetic variants influencing a broad range of traits [8]. However, recent work has proposed that pleiotropy is modular in nature, with specific loci affecting a limited subset of related traits [9,10]. This modularity opens the possibility of targeting specific genetic pathways for disease intervention while minimizing unwanted side effects.

Some studies focused on assessing the prevalence of pleiotropy in only small subsets of phenotypes [11,12] and others focused on the prevalence of pleiotropy in using all phenotypes in the entire GWAS catalog. Chesmore et al. [13] found that 44% of genes reported in the GWAS catalog were associated with more than one phenotype. Watanabe et al. [14] systematically analyzed more than 4000 publicly available GWASs, indicating the presence of widespread pleiotropy both at the levels of genes (63%) and Single Nucleotide Polymorphisms, SNPs (31%).

More recently, several studies have addressed the pleiotropy issue of human genes through different approaches [14]. Wang et al. [15] developed a new method to identify pleiotropic genes from GWAS summary statistics and applied it to 14 psychiatric disorders; they identified 32 genes associated with at least five psychiatric disorders. Islam et al. [16] investigated pleiotropic SNPs shared between various glycemic traits and migraine, and identified 20 genomic regions, along with 14 key SNPs. All of these SNPs, except one, are located in introns or intergenic regions. In the study by Barrio-Hernandez et al. [6], the authors adopted an original approach based on gene interaction networks involved in over 1000 human diseases to identify 73 modules of pleiotropic genes. They found that these modules were enriched with genes involved in RNA processing and protein ubiquitination. In a recent study, Qi et al. [4] analyzed 4114 traits from the UK Biobank GWAS data and assessed the level of pleiotropy for 2293 key SNPs. They identified 778 pleiotropic SNPs, including 58 highly pleiotropic SNPs associated with 16 or more diseases. In a very recent GWAS, Qiang et al. [17] identified 24,423 SNPs across 427 loci associated with the plasma concentration of 249 metabolites in nearly 255,000 individuals, of which 323 (75%) were pleiotropic.

In this study, we performed a large-scale analysis of the GWAS catalog to explore the extent and nature of pleiotropy in human complex diseases and identify key pleiotropic genes. Our approach is based on applying stringent criteria for selecting SNP variants that are associated to at least two disease phenotypes and on a composite score that identifies highly likely pleiotropic genes. The top genes identified were screened to understand further the functional role of the variants reported on the associated phenotypes.

## 2. Materials and Methods

### 2.1. Data Retrieval, Checking and Curation

The GWAS catalog [2] was downloaded on 2 January 2025. All SNPs that have been reported to be associated with at least two different traits in at least two independent studies were kept. The second filtering step considered keeping only SNP–trait associations reported with a significance level of *p*-value < 10^−10^. Finally, only SNP–trait associations involving binary traits were selected. Because our study is gene-centered, each SNP was assigned to the gene in which it is located (if intragenic) or to the nearest mapped gene(s) according to the GRCh38.p10 reference genome. Intergenic SNPs that were located far from any gene were discarded; only those within 2 kb of the nearest gene were retained. In cases where an intergenic SNP was positioned at a similar distance from two flanking genes, it was assigned to both.

A total of 494 putative pleiotropic genes were thus identified, corresponding to 223 phenotypes and 2895 gene–phenotype associations from the GWAS catalog. Phenotypes were manually curated to class binary traits into disease domains, according to the international classification of diseases and related health problems (ICD-10) tenth revision [18] (Organization World Health 2004). The trait-associated genes were further categorized into two groups, including (1) multidomain genes that were significantly associated with traits belonging to at least two domains and (2) domain-specific genes that were significantly associated with more than one trait from the same domain.

For each retained SNPs, Odds Ratios (ORs) were retrieved from the GWAS catalog (in the case where only Beta, the coefficient estimated from logistic regression, is reported, the OR was calculated as exp(beta)). SNPs for which no data on Beta or OR in the GWAS catalog were checked from the original publications and if no data were provided, were discarded. Associations from papers reporting metanalyses of GWASs were also discarded. Finally, SNPs with only one association (given that some SNP–trait associations have been removed because of missing Beta or OR, we might end with an SNP associated to a single trait) were finally removed.

The selection criteria, including the *p*-value threshold (<10^−10^), restriction to binary traits, and exclusion of meta-analyses, were chosen to ensure consistency and minimize heterogeneity across datasets. The stringent *p*-value threshold was set to reduce false positives in large-scale analyses, while focusing on binary traits allowed for clearer harmonization across cohorts.

*p*-values were transformed into Z-scores as recommended by Cox [19] for the treatment of small *p*-values. Z-scores were derived from estimating a normally distributed effect and provide an additional measure of the association signal: Z = Φ^−1^(1 − *p*), where Φ is the probability distribution function of the normal distribution and *p* the *p*-value.

The filtering process of data from the GWAS using the above-mentioned criteria was carried on by three independent persons (IM, GC, AR) and discrepant results were verified, and a consensus database was produced. A final manual check of the database, SNP by SNP, was performed by checking the reliability of the reported associations from the source papers.

The final dataset contains 343 SNP–trait association signals corresponding to 16 genes (see Table 1, results Section 3) and 53 SNPs. For each association signal, ten features were reported: the rs code of the SNP, the gene where the SNP is located in or attributed to (in case of intergenic variants), the disease domain, the disease trait, the OR, the Z-score, the number of cases and controls in the original study, and the location/type of the SNP (intron, UTR, missense, non-sense or frameshift variants).

**Figure 1:**
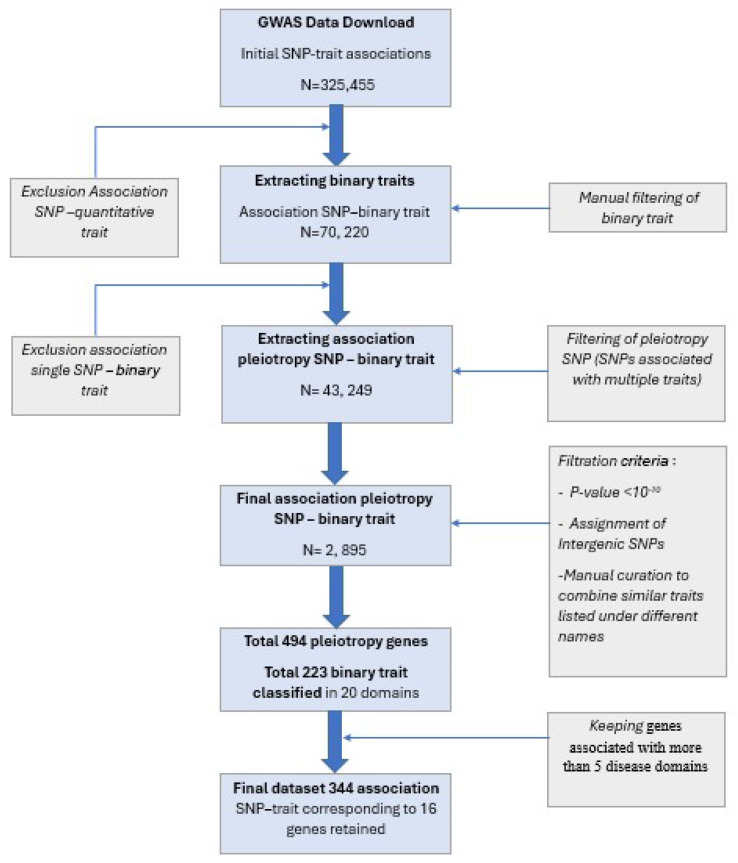
Flow diagram showing the pipeline for the selection of SNPs in pleiotropic genes.

Figure 1 summarizes the selection steps of pleiotropic SNP variants.

### 2.2. Data Analyses

The average association effect size for each gene was calculated according to the formula proposed by Chesmore et al. [13]:

(1) $${OR}_{g}=\frac{1}{n}[\sum_{i=1}^{n}\sum_{j=1}^{mi}\left|log({OR}_{ij})|/m_{i}\right)]$$

where *ORij* denotes the OR for SNP *j* and phenotype *i* for a given gene (*g*), *mi* is the number of association signals for phenotype *i*, and *n* the total number of association signals reported for the gene *g*.

A score-function was then used as a measure of the likelihood of a gene being pleiotropic. This score-function was calculated based on four elementary scores, defined as follows:

Association signals ($S_{g}$): Count of independent GWAS signals mapped to gene *g*.

Diseases ($D_{g}$): Count of distinct disease/trait categories associated with gene *g*.

Average OR (${OR}_{g}$) at the gene level calculated as described above.

Average Z-score ($Z_{g}$): Weighted average of the Z-scores of the association signals for gene *g*; since the studies have different sample sizes, we weighed by inverse variance, as follows (where $Z_{gi}$ is the Z-score for association signal *i* at gene *g* and $N_{i}$ is the total sample size of the study reporting this signal):

$$Z_{g}=\frac{\sum_{i=1}^{n}{N_{i}Z}_{gi}}{\sum_{i=1}^{n}N_{i}}$$

All these four elementary scores were normalized as follows:

$$S_{g}^{*}=\frac{log(1+S_{g})}{log(1+S_{max})};\;D_{g}^{*}=\frac{log(1+D_{g})}{log(1+D_{max})}$$

$${OR}_{g}^{*}=\frac{\left({OR}_{g}{-OR}_{min}\right)}{\left({OR}_{max}{-OR}_{min}\right)};\;Z_{g}^{*}=\frac{\left(Z_{g}{-Z}_{min}\right)}{\left(Z_{max}{-Z}_{min}\right)}$$

A composite score was then calculated as a weighted product of the four attributes, in a similar way to Multiplicative Weighting Method in multicriteria decision-making [19].

$${PS}_{g}={\left(S_{g}^{*}\right)}^{\alpha_{S}}\;{\left(D_{g}^{*}\right)}^{\alpha_{D}}{\left({OR}_{g}^{*}\right)}^{\alpha_{OR}}{\left(Z_{g}^{*}\right)}^{\alpha_{Z}}$$

The weights (α) were selected to achieve a balanced contribution across the four attributes, while placing greater emphasis on disease breadth and statistical strength. This prioritization ensures that genes associated with a wider range of independent GWAS signals and diverse disease categories are highlighted, and that their ranking is supported by robust statistical evaluation. At the same time, effect size was incorporated with moderate influence, so that it contributes meaningfully without overshadowing the breadth and statistical dimensions: $\alpha_{S}$ = 2, $\alpha_{D}$ = 2, $\alpha_{OR}$= 0.5, $\alpha_{Z}$ = 1. A sensitivity analysis showed that this combination of weights better discriminates between genes.

All data analyses and figures were generated using the R language (R Foundation for Statistical Computing, Vienna, Austria, https://www.r-project.org/).

The SNPs located in the pleiotropic genes identified in our study were investigated for their functional effect based on many resources including ClinVar, eQTL database, and in silico prediction tools: AlphaMissene, SpliceAI and RegSNP-intron [20,21,22]. The LDmatrix tool from the LDlink platform was used to assess linkage disequilibrium (LD) between SNPs within a given gene [23].

We declare here that generative artificial intelligence (GenAI) has not been used in this paper to generate text, data, or graphics, or to assist in study design, data collection, analysis, or interpretation.

## 3. Results

### 3.1. Number of SNP–Trait Associations per Gene and Disease

Our final database contains 53 SNPs, where the number of associations signals per SNP ranged from two to 25 (median 4). The SNP showing the largest number of associations is rs1260326, a missense variant located in the coding region of *GCKR*. The number of association signals (SNP–trait combination) per pleiotropic gene varies from four, for SORCS3 to 59, for TERT, with a median value of 20 (Table 1).

The number of associations per trait category varies from one for scoliosis to 82 for cancer (neoplasms), with a median of six. Cancer, cardiovascular, and endocrine diseases are the most represented with 82, 58 and 37 association signals, respectively.

### 3.2. Effect Size at the Gene Level

**Figure 2:**
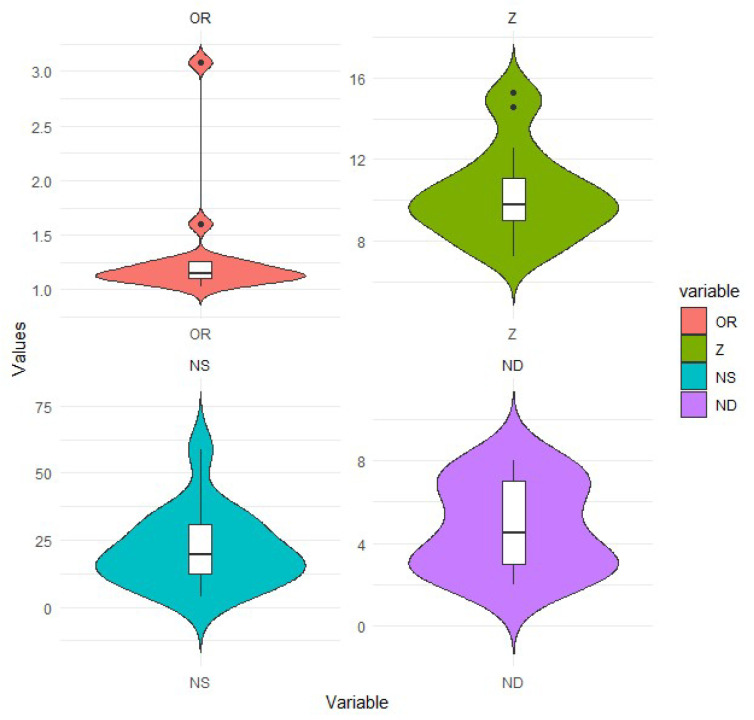
Violin plots of the four statistics used to characterize the level of pleiotropy of a gene (see text for details).

The effect size (OR) at the gene level shows a symmetric distribution, if we exclude the two outliers, ALDH2 and HLA-DQA1 (Figure 2) with an average 1.30 ± 0.49 and a median of 1.15 (IQR= 0.15); the maximal value was observed for HLA-DQA1 and the minimum of for SORCS3 (Table 1). It is worth noting that all SNPs (for all genes) show ORs that are in the range 1 to 1.5, except for the genes APOE and HLA-DQA1, where high ORs (>3) were reported by some studies (Figure S1).

There is no significant correlation (Pearson correlation *p*-value > 0.05) between any pair of the four metrics in Table 1, indicating that they are independent attributes of gene-level association signals and justify their combination in a multiplicative composite score.

The composite score shows a multimodal distribution with an average of 0.079 (Figure S2).

We used this average as the cutoff to identify the top pleiotropic genes, retaining four (highlighted in bold in Table 1). The TERT gene was additionally included, as its value was just below the threshold.

### 3.3. Type of Variants Within Pleiotropic Genes

Among the SNPs reported in our database, about 70% (37) are in introns, five in UTR, and 11 in coding regions, among which eight (15.8%) were missense variants, two were synonymous and one frameshift.

### 3.4. Pleiotropic Genes and Variants Within Pleiotropic Genes

#### 3.4.1. ABO Gene

The ABO gene encodes a glycosyltransferase, an enzyme that modifies the antigens present on the surface of red blood cells. This enzyme is responsible for adding specific sugar to the H antigen to form the A or B antigen. The gene is located on chromosome 9 (9q34.2), consists of eight exons, and spans 42 kb.

**Figure 3:**
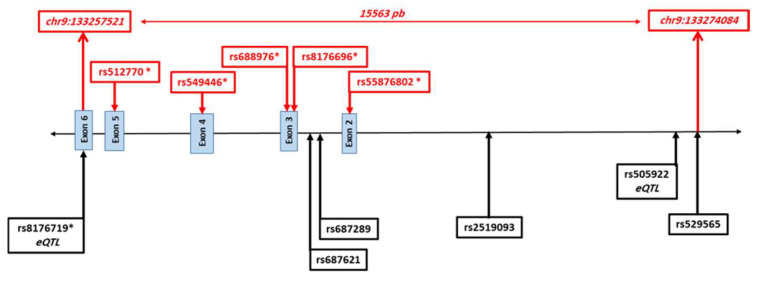
Schematic representation of ABO gene regions harboring pleiotropic SNPs identified. SNPs in black with a star indicate exonic frameshift variants, while red starred SNPs are pathogenic missense variants identified in ClinVar but absent from the GWAS catalog. An eQTL label is added when reported for the corresponding SNP.

Our results report 32 strong association signals with cardiovascular diseases, Type 2 diabetes, pancreatic cancer, malaria, and idiopathic scoliosis, corresponding to six SNPs extending over 15.5 kb, one of which is a frameshift variant, while the remaining five are located within introns (Figure 3). The prediction of potential effects of these intronic SNPs on gene expression or splicing yielded no significant findings (all were classified as benign or not reported in ClinVar).

The variant rs8176719 is a deletion polymorphism (c.261delG) located in exon 6 of the ABO gene. It causes a frameshift mutation, resulting in a truncated, non-functional protein (blood group O). It is particularly noteworthy that all six SNPs belong to the same linkage disequilibrium (LD) block, with r^2^ coefficients ranging from 0.75 to 0.98 (Figure S3), indicating that the causal SNP is most likely rs8176719. It is also possible that other missense variants located within the identified region play a functional role; in fact, by exploring the region we were able to identify five missense variants located in exons 2, 3, 4 and 5 (indicated in red in Figure 3).

#### 3.4.2. ALDH2 Gene

The ALDH2 gene encodes a mitochondrial enzyme, Aldehyde Dehydrogenase 2, which detoxifies acetaldehyde, a toxic compound produced during alcohol metabolism. The gene is located on chromosome 12 (12q24.12), consists of 13 exons, and spans 50 kb.

**Figure 4:**
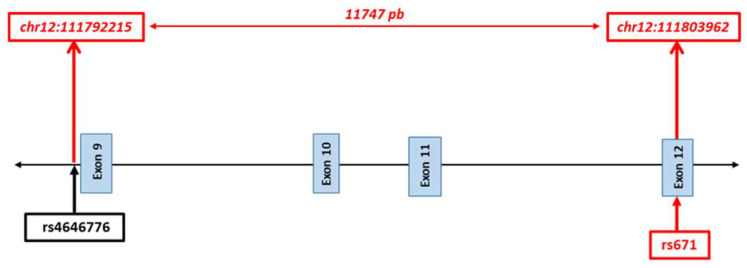
Schematic representation of ALDH2 gene regions harboring pleiotropic SNPs. Missense SNPs are indicated in red while non-coding SNPs are in black.

According to our analysis, 20 strong association signals with several diseases (psoriasis, cirrhosis, cardiovascular disease, esophageal cancer, autoimmune disease) were identified, corresponding to two SNPs: one intronic and one missense (Figure 4).

The SNP rs671, located in exon 12, has the largest number of associations, being linked to almost all diseases. This SNP is a functionally significant missense variant that changes position 504 of the protein (Glu504Lys, previously named E487K when excluding the N-terminal 17 residues corresponding to the mitochondrial leader peptide), is classified as pathogenic (Alpha Missense pathogenicity: 98.8%) and results in an inactive or weakly active enzyme.

The SNP rs4646776, located at the end of intron 8 (near exon 9), is in very strong linkage disequilibrium with rs671 (r^2^ = 0.977), indicating that the association observed in GWASs is very likely attributable to rs671, which would therefore be the lead causal SNP.

#### 3.4.3. GCKR Gene

**Figure 5:**
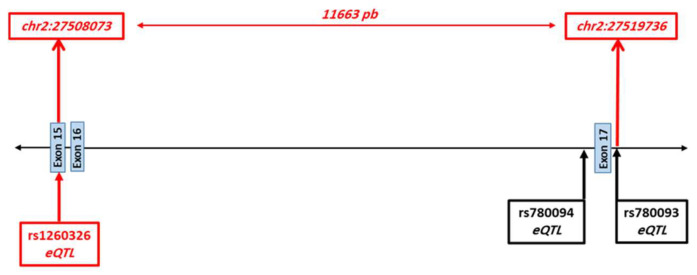
Schematic representation of GCKR gene regions harboring pleiotropic SNPs. SNPs in red are missense variants while noncoding SNPs are in black.

The GCKR gene (Glucokinase Regulator) encodes a regulatory protein of glucokinase (GCK). It is located on chromosome 2 (2p23.3), consists of 18 exons, and spans approximately 26 kb. We identified 29 strong association signals mainly with autoimmune diseases, metabolic disorders, Type 2 diabetes, and urinary diseases. These association signals correspond to three SNPs, two located in introns and one in an exon, covering a region of 11.7 kb (Figure 5). These SNPs belong to the same linkage disequilibrium (LD) block, with r^2^ values between 0.84 and 0.94.

The SNP with the greatest number of associations is rs1260326, located in exon 15, and associated with almost all the diseases reported. It is a functionally significant missense variant that changes position 446 of the protein (L446P: L > P, Q, R), influencing glucose and lipid metabolism. Although it is classified as benign in the ClinVar database, it is predicted to be likely pathogenic by AlphaMissense (score: 88.9%). The two other SNPs (located in introns 16 and 17) have no direct effect on gene expression and both are classified as benign or not reported in ClinVar.

eQTL data analysis revealed that rs1260326 and rs780093 correspond, in ClinVar, to QTL5 for fasting blood glucose and blood lipid levels [19]. They are also significantly associated with the expression of the gene in skeletal muscle tissues.

#### 3.4.4. HLA-DQA1

The HLA-DQA1 gene (Human Leukocyte Antigen DQ Alpha 1) encodes the alpha chain of a protein that associates with a beta chain (encoded by the HLA-DQB1 gene) to form a DQ heterodimer, a surface glycoprotein involved in antigen presentation and playing a central role in the adaptive immune system. The HLA-DQA1 gene, part of the major histocompatibility complex (MHC) class II, is located on chromosome 6 (6p21.31), contains five exons, and spans 6 kb.

**Figure 6:**
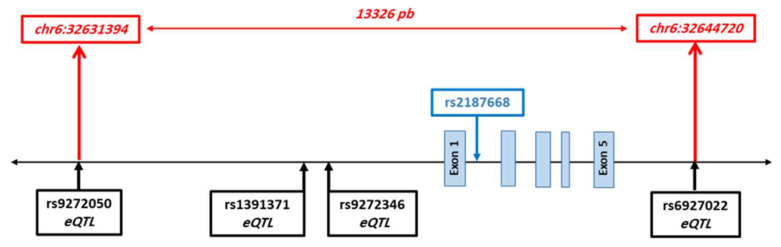
Schematic representation of HLA-DQA1 gene regions containing pleiotropic SNPs. SNPs in black indicate non-coding variants, and eQTL indicates that the SNPs mapped on an expression QTL.

In our study, we identified 19 strong association signals at this gene with several diseases: gastrointestinal, immunological (celiac disease, type 1 autoimmune hepatitis, systemic lupus erythematosus, gastric ulcer, gastroesophageal reflux), respiratory (asthma), dermatological (eczema), cervical cancer, and Type 1 diabetes. These associations correspond to four SNPs, all intronic, covering a region of more than 13 kb (Figure 6).

Three of these four SNPs are in the UTR regions and are likely involved in regulating gene expression. They show moderate levels of linkage disequilibrium, with r^2^ values ranging from 0.12 to 0.38. The SNPs rs9272050 and rs6927022 are reported in ClinVar without any information on their clinical significance and do not appear in any publication.

#### 3.4.5. TERT Gene

The TERT gene (telomerase reverse transcriptase) encodes the catalytic subunit of the telomerase enzyme, whose main function is to maintain telomere length. Telomeres are highly repetitive, non-coding DNA sequences located at the ends of each chromosome, playing a role in protecting them from degradation during aging. The gene is located on chromosome 5 (5p15.33), consists of 16 exons, and spans 41 kb.

TERT gene has the highest number of association signals in the GWAS catalog (59) across seven different cancers (breast, prostate, ovarian, pancreatic, lung, skin, and glioma), in addition to associations with pulmonary and uterine fibrosis. These association signals correspond to nine SNPs, eight of which are in introns and one in the coding region (although synonymous).

**Figure 7:**
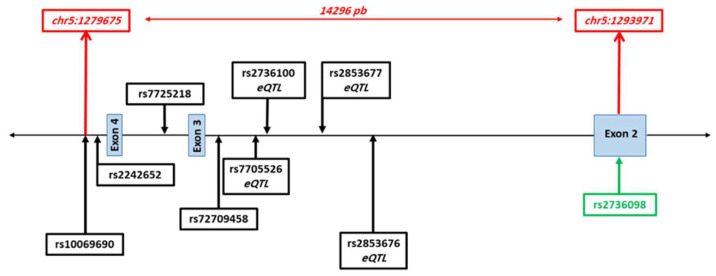
Schematic representation of TERT gene regions containing pleiotropic SNPs. SNPs shown in green represent coding synonymous variants, while those in black indicate other types. An eQTL comment is included when an eQTL has been reported for the corresponding SNP.

The SNP with the largest number of associations is rs10069690, linked to four different cancers. Functional annotations of these eight variants (located in introns 2, 3, and 4) using various prediction tools showed that none of them has a direct effect on gene expression (all classified as benign or not reported in ClinVar). These eight SNPs cover a region of 8.7 kb (Figure 7), going from rs10069690 to rs2853676, and belong to the same linkage disequilibrium block with r^2^ values ranging from 0.2 to 0.8 (Figure S4).

eQTL analysis showed that SNP rs10069690 is associated with altered expression of this gene. This association was observed in human induced pluripotent stem cells (iPS cells). Four other variants correspond to eQTLs and are associated with gene expression in skin tissue (Figure 7).

## 4. Discussion

Our study of the GWAS catalog allowed us to identify 53 key SNPs reported as associated with multiple diseases, which we considered pleiotropic. However, since pleiotropy refers to a gene acting on multiple different phenotypes, we focused our analysis on the genes in which these SNPs are located, and identified 16 genes, five of which were selected based on a composite score and were further investigated to reveal the functional role and clinical impact of the reported variants.

One of the first studies on pleiotropy using the GWAS catalog was published in 2011 by Sivakumaran et al. and highlighted 233 genes and 76 SNPs. The localization of these SNPs indicated that only 14.5% are in coding regions, while 35.5% are intronic and 15.8% are in intergenic regions. Among their pleiotropic genes, we identified 10 that also appear in our study, namely ALDH2, APOE, ATXN2, IRF4, JAZF1, GCKR, HLA-DQA1, SH2B3, STAT4, and TERT. In a subsequent study, also based on analysis of the GWAS catalog, Chesmore et al. [13] identified eight leading genes by applying criteria of comparable stringency to those employed in our study. Only the genes ABO and GCKR are common between their list and ours. In their study of cross-population variants associated with 220 phenotypes, Watanabe et al. [14] revealed variants, which we also identified in our study, namely rs671 (ALDH2, 47 traits), rs1260326 (GCKR, 20 traits), rs7310615 (ATXN2/SH2B3, 38 traits), rs3184504 (SH2B3), and rs2519093 (ABO, 28 traits), which rank among the top pleiotropic variants.

Wang et al. [24] developed a method to identify pleiotropic genes from GWAS data and tested it on behavioral diseases. They identified 32 genes, many of which correspond to microRNA or long non-coding RNA (lncRNA) genes. Only GCKR is common with our list. In another recent study, Islam et al. [16] examined pleiotropic SNPs shared between different glycemic traits and migraine, identifying 20 genomic regions including one with the ABO gene and 14 key SNPs. All these SNPs, except one, are in introns or intergenic regions.

Barrio-Hernandez et al. [6] adopted an original approach based on gene interaction networks involved in more than 1000 human diseases to identify 73 modules of pleiotropic genes. They found that these modules were enriched in genes involved in RNA processing and protein ubiquitination. Among the key genes identified, five overlap with our list: HLA-DQA1, IRF4, SLC39A8, SMAD3, and STAT6.

In a recent study, Qi et al. [4] analyzed 4114 traits from UK Biobank GWAS and assessed the level of pleiotropy for 2293 key SNPs. They identified 778 pleiotropic SNPs, including 58 highly pleiotropic SNPs associated with 16 or more diseases. Among the main genes identified as pleiotropic, we find SH2B3 (rs3184504), GCKR (rs1260326), ABO (rs687289), and genes in the HLA region (notably HLA-DQB1). They also found that pleiotropic SNPs are more likely to be in regulatory regions of the genome (enhancers, promoters, UTRs), are subject to strong evolutionary pressure, and several are near or within genes that are known drug targets. Regarding SNPs, only two SNPs are common between our database and their list of 778 pleiotropic SNPs: rs738408 (PNPLA3) and rs7705526 (TERT).

In a very recent GWAS, Qiang et al. [17] identified 24,423 SNPs in 427 loci associated with plasma concentration of 249 metabolites in nearly 255,000 individuals, of which 323 (75%) were pleiotropic. Of these SNPs, eight overlap with our list of pleiotropic variants: rs2519093 (ABO), rs671 (ALDH2), rs769449 (APOE), rs3184504 (SH2B3), rs13107325 (SLC38A9), rs17293632 (SMAD3), rs3122929 (STAT6), and rs2736100/rs2853676 (TERT).

It is interesting to note that four of the pleiotropic genes identified in our study consistently reappear in most of the previously cited studies, namely ABO, GCKR, SH2B3, and TERT. ABO is the pleiotropic gene with the highest score (highest gene effect and second largest number of association signals). The reported signals correspond to six SNPs, five of which are intronic and one frameshift. All six SNPs belong to the same linkage disequilibrium block, which could indicate that the causal SNP is variant rs8176719 (c.261delG in exon 6), resulting in a truncated, non-functional protein (blood group O). It is well established that blood group O is associated with a reduced risk of cardiovascular disease and venous thrombosis, whereas carriers of blood groups A, B, and AB have an increased risk of certain cancers (stomach, pancreas). Previous studies identifying the ABO gene as pleiotropic have reported SNPs rs2519093 and rs687289, located in introns 1 and 2, respectively. Variant rs2519093 is especially noteworthy, as it has been associated, independently of rs8176719, with venous thrombosis risk, and has been recommended (along with rs8176719 and two other SNPs) for inclusion in thrombosis risk estimation [25]. SNP rs687289 has been associated with the regulation of circulating protein levels implicated in inflammation [26]. It is therefore highly likely that these two intronic SNPs have important regulatory effects on ABO gene expression, and that their associations with disease phenotypes are not solely due to strong linkage disequilibrium with the frameshift variant rs8176719.

The association signals of ALDH2, mostly related to the digestive system, strongly indicate that the missense SNP rs671 (E504K) is the causal variant. This variant corresponds to the substitution of a negatively charged residue (Glutamate) with a positively charged one (Lysine), thereby altering the local charge of the protein in the C-terminal region around position 504 (the ALDH2 protein has a total of 517 residues). Residue E504 is involved in the structural stability of the ALDH2 tetramer by forming a hydrogen bond with an Arginine at position 492 of the other monomer (critical for dimer stability) and in substrate binding (acetaldehyde, for which it has high affinity), which occurs via a coenzyme NAD+ binding pocket and a Cysteine at position 302 [27]. Its substitution by a Lysine results in partial or complete inactivation of ALDH2, leading to accumulation of acetaldehyde in the blood of carriers of the K allele, especially in the homozygous state (allele ALDH2*2 according to PharmGKB nomenclature). This allele is extremely rare (<0.001) in all populations, except in Asia (12–20%), particularly East Asia, where its frequency reaches 23% or more (up to 50% in Mongolia, Vietnam, and Laos).

A recent synthesis of several meta-analyses suggests that this variant, in addition to its well-known association with risk of stomach cancer diseases linked to high alcohol consumption, is also associated with increased risk of hypertension and cardiovascular disease, diabetes, and neurodegenerative diseases (such as Alzheimer’s and Parkinson’s) [28], particularly in East Asia. The mechanisms underlying the functional effect of this variant are often complex due to compensatory aldehyde detoxification systems and the fact that pathological phenotypes are influenced by interacting environmental factors (e.g., alcohol consumption and smoking) or other individual vulnerabilities (such as aging and the apolipoprotein E ε4 allele) [27]. Whatever the molecular and cellular pathways, rs671 is considered an important element in preventive medicine; carriers of the ALDH2*2 allele are advised to reduce their alcohol consumption to lower the risk of cancer and aging-related diseases (cardiometabolic and cognitive).

For the GCKR gene, the SNP with the largest number of associations is the missense variant rs1260326 (P446L), which reduces inhibition of glucokinase, thereby increasing its activity in the liver and leading to enhanced hepatic glycolysis and triglyceride production [29,30]. A recent study in a sample of 4890 Chinese individuals associated it with increased triglycerides and decreased HDL cholesterol, as well as insulin resistance and impaired pancreatic beta-cell function [31]. Indeed, the P446L substitution, although located in the C-terminal region, far from the catalytic site, alters the conformation of the GCKR protein and affects its ability to bind fructose-6-phosphate and regulate glucokinase activity. In addition, the two intronic SNPs rs780093 and rs780094, located on either side of exon 17 at 1367 bp from each other, have been found to be associated with low HDL-C levels and a 20% increased risk of cardiometabolic disease in a sample of 5666 Iranian individuals. More specifically, carriers of the TTT haplotype in the homozygous state for the three pleiotropic SNPs identified in our study are at increased risk [32].

The HLA-DQA1 gene is located between the HLA-DQA2 gene (35 kb upstream) and the HLA-DQB1 gene (20 kb downstream), with which it forms a heterodimer necessary for antigen presentation to CD4+ T cells. This gene (or its neighboring genes) was identified as pleiotropic in our study, with the strongest gene effect. The study by Qi et al. [4] identified HLA-DQB1 rather than HLA-DQA1 as pleiotropic. Indeed, the association signals we identified correspond to four SNPs, three of which are located between HLA-DQA2 and HLA-DQA1, and one in the last intron of HLA-DQA1, close to HLA-DQB1. The intronic SNP rs6927022 (intron 5) is mentioned in 12 studies, the most recent [33] reporting its strong association with antibody levels against the EBV virus. EBV infection is well known to be the cause of several autoimmune diseases and cancers [34,35]. This could provide an interesting lead for understanding the pleiotropic effect of this variant. The SNP rs2187668, located in intron 1 of the gene, although not reported in our database, is a marker of the HLA-DRB1*0301 allele, which has been associated with increased risk of autoimmune diseases [36]. Although the functional effect of this SNP is not known, it has been suggested that it may alter gene expression or splicing.

The TERT gene, which encodes the catalytic subunit of telomerase—an enzyme essential for maintaining telomere length—is, according to our analyses, the gene showing the highest number of GWAS association signals, particularly with various types of cancer affecting seven different organs. Interestingly, the eight SNPs that have been identified are intronic (introns 2 to 4) and span a region of 8.7 kb belonging to a single linkage disequilibrium block. Although variant rs10069690, located in intron 4, is identified in ClinVar as benign or with weak evidence for pathogenicity, the T allele of this variant is, according to the study by Florez-Vargas et al. [37], linked to increased retention of intron 4 in the TERT transcript. This retention results in an alternative mRNA form, which could reduce telomerase activity or alter its regulation. This variant has also been associated with increased risk of multiple cancers and age-related telomere shortening. By regulating TERT splicing, it may contribute to fine-tuning cellular longevity and replicative potential, particularly in response to stress induced by endogenous and exogenous tissue-specific exposures, thereby influencing cancer risk at this locus.

Song et al. [38] studied the association of five SNPs within the TERT gene and demonstrated, through Mendelian Randomization Trials (MRTs), that three of them (rs2853676, rs2242652, and rs2075786) have a causal effect on leukocyte telomere length in peripheral blood and on the risk of cerebral small vessel disease, a group of disorders affecting small arteries, arterioles, capillaries, and venules in the brain, and constituting a major cause of lacunar strokes and age-related cognitive decline. SNPs rs2853676 (intron 2) and rs2242652 (intron 4) are among the eight SNPs we identified, whereas rs2075786 is located at the beginning of intron 2, nearly 22 kb away from rs2853676 (which is near the end of the intron). This variant (rs2075786) has been associated with differential cancer risk in Lynch syndrome patients carrying mutations in the MSH2 gene and may act as a risk modifier [39]. The molecular mechanisms of this interaction remain to be clarified. Intron 2 of the TERT gene, where most of the pleiotropic SNPs identified in our study are located, is particularly long (22.2 kb) and may contain transcriptional regulatory elements: enhancers, transcription factor binding sites, or epigenetic elements influencing TERT expression. Moreover, many studies have shown that introns located near gene promoters are more frequently involved in transcriptional regulation than distal introns [40,41]. In addition, a recent study reported the involvement of two mutations in the TERT promoter: rs1242535815 (C228T, −124 bp from ATG) and rs1242535816 (C250T, −148 bp from ATG) in the resistance to thyroid cancer treatments and poor prognosis [42]. These variants result in the creation of new transcription factor binding sites, leading to overexpression of the TERT gene.

Our study is exploratory in nature and does not aim to experimentally validate biological pleiotropy but rather to identify candidate pleiotropic genes through robust statistical evidence and functional predictions. In several cases, functional studies and eQTL annotations already support pleiotropic effects, and some genes and variants have been experimentally examined, with regulatory roles of intronic variants established and cited where available. While replication of associations across independent samples provides strong evidence of biological significance, experimental validation remains beyond the scope of this work. The stringent filtering criteria applied further strengthen confidence that our top-ranked genes are unlikely to represent false positives.

## 5. Conclusions

Our study highlights five pleiotropic genes, which consistently reappear across previous research, underscoring their central role in complex disease associations. Key variants such as rs8176719 in ABO, rs671 in ALDH2, rs1260326 in GCKR, and intronic SNPs in TERT demonstrate strong functional and clinical relevance, linking genetic regulation to cardiometabolic, inflammatory, and cancer-related outcomes. These findings emphasize the importance of integrating genetic, functional, and population-level evidence to better understand pleiotropy and its implications for preventive medicine.

## Figures and Tables

**Table 1:** List of 16 pleiotropic genes identified and their relevant statistics.

Gene	Phenotypes	Signals	Dg* ^a^	Sg* ^a^	Zg ^a^	Org ^a^	Score ^b^
ABO	6	32	0.885	0.854	11.47	1.085	**0.088**
ACAD10	5	9	0.815	0.562	10.37	1.263	0.050
ALDH2	8	20	1	0.743	9.14	1.602	**0.157**
APOE	3	36	0.631	0.882	9.85	1.258	0.066
ATXN2	8	20	1	0.743	8.12	1.082	0.042
GCKR	7	31	0.946	0.846	9.52	1.138	**0.094**
HLA-DQA1	7	19	0.946	0.731	12.55	3.08	**0.407**
IRF4	3	16	0.631	0.692	15.27	1.257	0.069
JAZF1	2	13	0.5	0.644	10.67	1.156	0.019
PNPLA3	3	8	0.631	0.536	7.80	1.163	0.010
SH2B3	7	21	0.946	0.755	8.67	1.199	0.073
SLC39A8	3	14	0.631	0.661	14.56	1.087	0.035
SMAD3	5	31	0.815	0.846	9.75	1.124	0.069
SORCS3	3	4	0.631	0.393	7.22	1.024	0.001
STAT6	2	10	0.5	0.585	10.95	1.117	0.014
TERT	4	59	0.732	1	9.30	1.141	***0.076***

^a^: see text for the definition and formula to calculate these statistics. ^b^: in bold genes with above-average score; in bold italic, the gene with close to average score.

## Data Availability

The raw data supporting the conclusions of this article will be made available by the authors on request.

## Supplementary Materials

The following supporting information can be downloaded at: https://www.mdpi.com/article/10.3390/genes17070766/s1. Figure S1. Boxplot of the Odds Ratios (OR) calculated at the gene level for the 16 genes identified as pleiotropic in our study. Figure S2: Violin plot showing the distribution of the composite Score for all Pleiotropic genes identified in our study. Figure S3. Linkage disequilibrium (LD) between SNP identified as pleiotropic in the ABO gene (generated by LDmatrix tool). Figure S4. Linkage disequilibrium (LD) between SNP identified as pleiotropic in the TERT gene (generated by LDmatrix tool).

## Abbreviations

The following abbreviations are used in this manuscript:

GWAS	Genome-Wide Association Studies
SNP	Single Nucleotide Polymorphism
UTR	UnTranslated Region
LD	Linkage Disequilibrium
eQTL	Expression Quantitative Trait Locus
OR	Odds Ratio
