# Supplementary material for: Large-Scale Analyses of GWAS Identify Five Key Pleiotropic Genes Involved in Complex Diseases
Source: Genes (Basel). 2026 Jun 30;17(7):766. doi: 10.3390/genes17070766 (PMC13409521; doi:10.3390/genes17070766)
Supplement: Supplementary file 1 [file genes-17-00766-s001.zip › FigureS1.pdf]

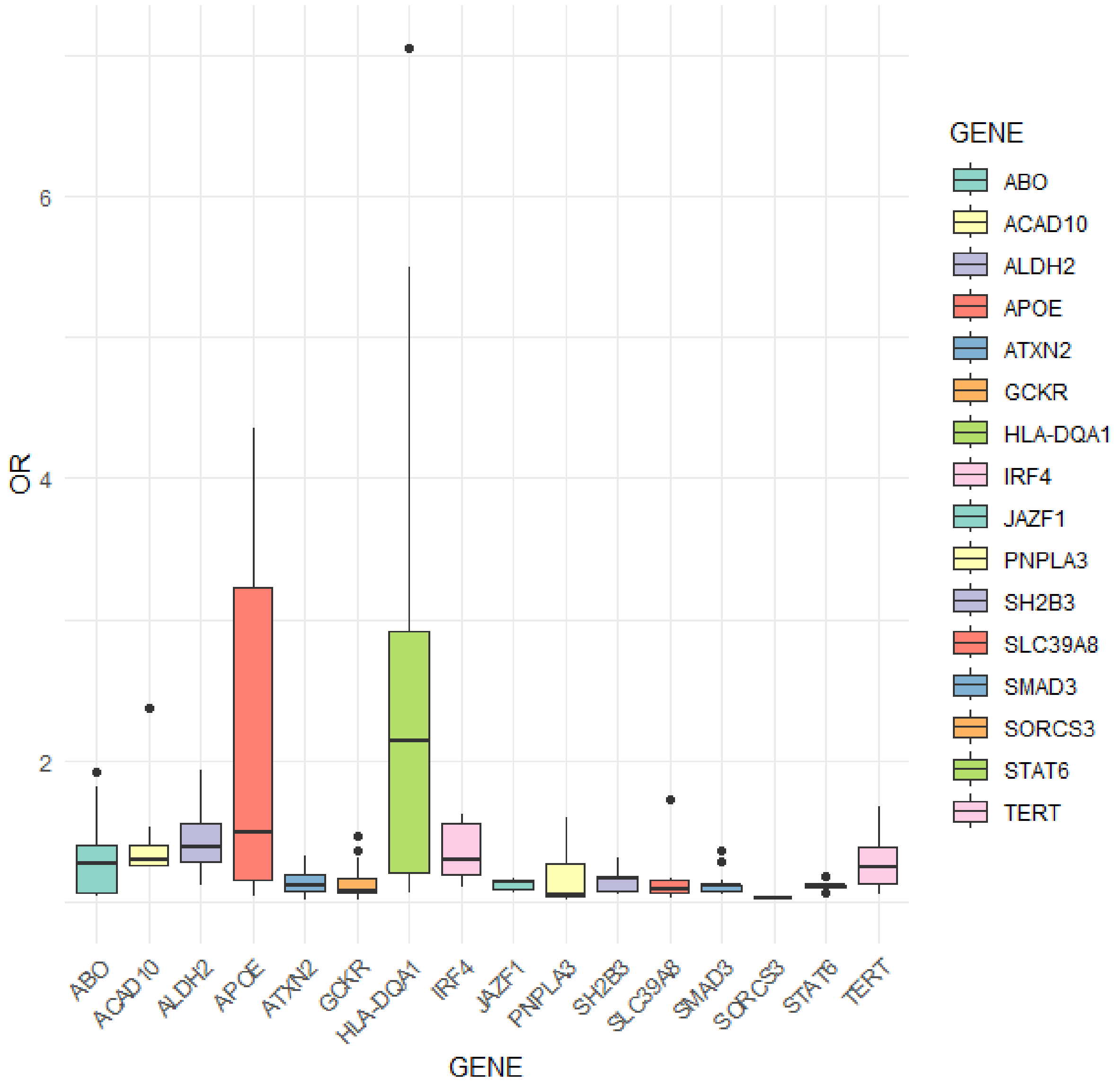

Figure S1. Boxplot of the Odds Ratios (OR) calculated at the gene level for the 16 genes identified as pleiotropic in our study.
